# The Primate EAE Model Points at EBV-Infected B Cells as a Preferential Therapy Target in Multiple Sclerosis

**DOI:** 10.3389/fimmu.2013.00145

**Published:** 2013-06-13

**Authors:** Bert A. ‘T Hart, S. Anwar Jagessar, Krista Haanstra, Ernst Verschoor, Jon D. Laman, Yolanda S. Kap

**Affiliations:** ^1^Department of Immunobiology, Biomedical Primate Research Centre, Rijswijk, Netherlands; ^2^Multiple Sclerosis Center, Erasmus MC, Rotterdam, Netherlands; ^3^Department of Neuroscience, University Medical Center Groningen, Groningen, Netherlands; ^4^Department of Immunology, Erasmus MC, University Medical Center Rotterdam, Rotterdam, Netherlands; ^5^Department of Virology, Biomedical Primate Research Centre, Rijswijk, Netherlands

**Keywords:** MS, EAE, EBV, non-human primate, B cell, T cell, immunotherapy

## Abstract

The remarkable clinical efficacy of anti-CD20 monoclonal antibodies (mAb) in relapsing-remitting multiple sclerosis points at the critical involvement of B cells in the disease. However, the exact pathogenic contribution of B cells is poorly understood. In this publication we review new data on the role of CD20+ B cells in a unique experimental autoimmune encephalomyelitis (EAE) model in common marmosets (*Callithrix jacchus*), a small-bodied neotropical primate. We will also discuss the relevance of these data for MS. Different from rodent EAE models, but similar to MS, disease progression in marmosets can develop independent of autoantibodies. Progressive disease is mediated by MHC class Ib (Caja-E) restricted cytotoxic T cells, which are activated by γ-herpesvirus-infected B cells and cause widespread demyelination of cortical gray matter. B-cell directed monoclonal antibody therapies (anti-CD20 versus anti-BLyS and anti-APRIL) have a variable effect on EAE progression, which we found associated with variable depletion of the Epstein Barr virus (EBV)-like γ-herpesvirus CalHV3 from lymphoid organs. These findings support an important pathogenic role of CD20+ B cell in MS, especially of the subset infected with EBV.

## Introduction

Multiple sclerosis (MS) is generally viewed as a neuroinflammatory disease initiated by a CD4+ T cell led autoimmune attack on the central nervous system (CNS), culminating into inflammation and tissue injury (Sospedra and Martin, 2005). Although the cause of MS is not known, infection of genetically susceptible individuals with (as yet unidentified) pathogen(s) is considered a likely trigger of CNS targeting cellular and humoral autoimmune reactions. Once induced, autoimmunity against myelin and non-myelin antigens drives progression of the disease to irreversible neurological deficit.

The prevailing concepts of MS pathogenesis are strongly influenced by the experimental autoimmune encephalomyelitis (EAE) animal model. However, although the relevance of the EAE model for our understanding of the autoimmune processes in MS has been enormous, the translation of EAE-based pathogenic concepts to the MS patient has often failed. The conceptual gap between the EAE model and MS is probably best illustrated by new treatments that fail to reproduce promising beneficial effects observed in the EAE model when they were tested in MS patients.

A well-recognized complication in preclinical therapy development is the immunological gap between inbred/SPF raised mouse strains used for MS modeling and the genetically and microbiologically heterogeneous patient population (Sachs, 2003; Davis, 2008). This probably also explains why certain pathogenic aspects of MS are not recapitulated in classical mouse EAE models – including the conversion from relapsing-remitting to progressive disease, a presumed prominent pathogenic role of CD8+ T cells and demyelination in cortical gray matter. The question is warranted whether these characteristic features of MS progression may be more accurately replicated in species that are more closely related to humans.

In this review we will present new data from immunopathogenesis and immunotherapy studies in a well-validated non-human primate EAE model that can help bridge the gap as it shares important similarities with rodent EAE as well as with MS. After briefly reviewing the role of T cells in the models, we will focus the discussion on a mechanistic explanation for the remarkable efficacy of B-cell depletion with anti-CD20 monoclonal antibodies (mAbs) (reviewed in Barun and Bar-Or, 2012) and for the still elusive association of Epstein Barr Virus (EBV) infection with MS (reviewed in Ascherio and Munger, 2007; Lunemann et al., 2007).

## Animal Models of MS

Multiple sclerosis researchers have only limited possibilities for investigating in patients immunological mechanisms that drive the disease and the ensuing pathological events within brain and spinal cord. Animal models have therefore been very important for the development of pathogenic and therapeutic concepts (reviewed in Ransohoff, 2012). The mouse is the most frequently chosen model for translational research into MS. A plethora of experimental models is now available, although only a limited number of susceptible strains (C57BL/6, SJL, BALB/c, Biozzi ABH) is used.

As reviewed in more detail elsewhere, clinical and pathological aspects of MS can be elicited via virus infection, by active immunization or by passive transfer of autoreactive T cells (reviewed in ‘T Hart et al., 2011). More sophisticated are the models in transgenic mice where MS-like pathology and disease develop spontaneously (Krishnamoorthy et al., 2007; ‘T Hart et al., 2011). Although mouse models have been extremely valuable for our understanding of neuroimmune deviations in MS, the translation of new pathogenic concepts into effective treatments for MS has been notoriously difficult thus far (Steinman and Zamvil, 2006; ‘T Hart et al., 2011). This has triggered criticism on the relevance of EAE model as a preclinical model of MS (Sriram and Steiner, 2005; Ransohoff, 2006, 2012). Nevertheless, several effective treatments, natalizumab for example, are directly based on preclinical research in this model (Steinman, 2005).

Throughout the years, the EAE model in common marmosets, a small-bodied neotropical primate, has been important for the efficacy testing of biological treatments, which due to their species-specificity cannot be tested in the more widely used rodent EAE models (‘T Hart and Bajramovic, 2008; ‘T Hart et al., 2011).

## The Elusive Role of B Cells

The clinical efficacy of systemic B-cell depletion by anti-CD20 mAbs in relapsing-remitting multiple sclerosis (RRMS) was rather unexpected, as literature data on the exact role of B cells in EAE and MS are confusing and sometimes paradoxical. The traditional role of B cells in widely adhered EAE-based autoimmune concepts of MS is considered less prominent than the role of pro-inflammatory Th1/Th17 cells, namely the production of autoantibodies that mediate injury to axon enwrapping myelin sheaths (demyelination) via complement- and macrophage-dependent cytotoxicity reactions (Krumbholz et al., 2012). However, experiments in transgenic mice show that autoreactive T and B cells are both needed for the induction of full-blown demyelinating disease (Pollinger et al., 2009). On the other hand, EAE studies in mice (Oliver et al., 2003) as well as non-human primates (NHP) (Jagessar et al., 2010) showing that autoimmune demyelination of CNS white matter can be induced independent of autoantibodies, warrant questions on the exact pathogenic role of B cells.

Therapeutic mAbs that target both the T- and B-cell arms of the autoimmune attack show promising effects in RRMS. Examples are alemtuzumab, a depleting mAb against CD52 expressed on B cells, T cells and monocytes (Klotz et al., 2012), and natalizumab, a blocking mAb against α4β1 integrin that blocks the CNS immigration of these cell types (Engelhardt and Kappos, 2008). However, mAbs that preferentially target pro-inflammatory CD4+ T helper (Th)1 cells such as anti-CD4 mAb (Racadot et al., 1993; Lindsey et al., 1994; van Oosten et al., 1996) or that interfere with the differentiation of antigen-activated CD4+ T cells toward Th1, such as the anti-IL-12p40 mAb ustekinumab, have shown only moderate or no effects (Segal et al., 2008). By contrast, selective depletion of B cells via mAbs against the B lineage specific surface marker CD20 (rituximab, ocrelizumab, and ofatumumab) proved remarkably effective in the induction of long-lasting suppression of lesion activity and clinical relapses (Barun and Bar-Or, 2012). On the other hand, induction of peripheral B-cell depletion via a soluble receptor of the TACI receptor (atacicept) for the B-cell cytokines BlyS (B lymphocyte stimulator; also known as BAFF) and APRIL (A Proliferation Inducing Ligand) did not suppress disease activity in RRMS (Hartung and Kieseier, 2010). Rather unexpectedly suppression of lesion activity and/or clinical symptoms by anti-CD20 mAb was not consistently associated with a reduction of serum autoantibody levels (Krumbholz et al., 2012).

In conclusion, although results from therapy trials in RRMS seem to confirm the important pathogenic role of T as well as B cells, they conflict with the classical concept that MS is caused by a combined autoimmune attack by Th1 cells causing inflammation and autoantibody producing B cells causing demyelination. How can this paradox be explained?

We will review here new data on the pathogenic role of CD20+ B cells in the unique EAE model in common marmosets. The model closely resembles MS in its immunological complexity and its clinical and pathological presentation (‘T Hart and Massacesi, 2009) and is therefore particularly suitable for translational research into pathogenic mechanisms and the influence of genetic and environmental factors. After a concise summary of the cellular autoimmune events that drive disease initiation and progression in the model, we will zoom in on the role of B cells. The dissection of the model into its main pathogenic components in combination with efficacy analysis of B-cell targeting therapeutic mAbs, has led us to postulate that the activation of CNS myelin specific cytotoxic T cells, which have a core pathogenic role in the chronic phase of the model (‘T Hart et al., 2011), is mediated by γ-herpes virus-infected B cells.

## Concise Overview of the Marmoset EAE Model

The central difference between EAE models in immunologically naïve inbred/SPF rodents and in the adult outbred conventionally housed NHP is that the disease in NHP develops in an immune environment that is shaped by genetic diversity and the daily combat with new and opportunistic infections. The consequences of the more human-like genetic, immunological, and microbiological condition of NHP for the clinical and pathological presentation of EAE have been extensively studied in the common marmoset (‘T Hart and Massacesi, 2009).

In brief, we found that among the complex patterns of autoimmune reactions developing in marmosets sensitized against CNS myelin, T- and B-cell responses against the quantitatively minor myelin oligodendrocyte glycoprotein (MOG) are critical for the development of chronic EAE (Jagessar et al., 2008).

The subsequently developed EAE model in marmosets, induced with recombinant human MOG_1–125_ (rhMOG) formulated with complete Freund’s adjuvant (CFA), can be characterized as “half mouse, half man.” The two-faced character of the model is reflected by the existence of two non-overlapping pathogenic mechanisms, a *mouse EAE-like initiation pathway* and *a human MS-like progression* pathway (see summary in Table 1) (‘T Hart et al., 2011).

**Table 1:** **Characteristics of the two autoimmune pathways proposed to elicit clinically evident EAE in marmosets immunized with rhMOG/CFA**.

	Pathway 1	Pathway 2
**Main activity**	EAE induction	EAE progression
**Immune signature**
T cell mediator	Anti-MOG_24–36_ Th1	Anti-MOG_40–48_ NK-CTL role Th17 cells uncertain
MHC restriction	Caja-DRB *W1201	Caja-E
Suppressed by	Anti-IL-12p40 mAb	Anti-CD20 mAb
	Anti-CD20 mAb	
	Anti-CD40 mAb	
**Selectively inducible**	MOG14–36/CFA	MOG34–56/IFA
**Pathology**	Inflammation of white matter	Inflammation and demyelination of white and gray matter

*A detailed discussion of the two pathways is provided in ‘T Hart et al. (2011). Anti-CD40 is an antagonist antibody blocking APC-T cell interaction*.

### The EAE initiation pathway

Just like in classical mouse EAE models, immunization of marmosets with rhMOG formulated with CFA induces the activation of Th1 cells and autoantibodies. The Th1 cells are specific for the epitope MOG_24–36_ and activation is restricted by a monomorphic MHC class II allele (Caja-DRB1^∗^W1201). Th1 cells induce inflammation in the white matter of brain and spinal cord (Brok et al., 2000; Doxiadis et al., 2006; Kap et al., 2008). Demyelination is also induced, but is mediated by autoantibodies that bind conformationally intact MOG and elicit cytotoxicity by macrophages (ADCC) or complement (CDC). The separation of effector mechanisms is illustrated by the finding that demyelination in marmosets immunized with MP4, a chimeric protein composed of myelin basic protein (MBP) and proteolipid protein (PLP), developed only in monkeys that formed anti-MOG antibodies (McFarland et al., 1999). Moreover, it was shown that adoptive transfer of an anti-MOG21–40 Th1 cell clone (Villoslada et al., 2001) induced only white matter inflammation and that demyelination in monkeys immunized with MBP or PLP could be induced by adoptive transfer of a monoclonal antibody (mAb) against rat MOG (8.18.C5) (Genain et al., 1995).

The finding that early treatment with the anti-IL-12p40 mAb ustekinumab completely abrogates EAE development illustrates the pathogenic relevance of the mouse EAE-like initiation pathway (‘T Hart et al., 2005). However, when the treatment with ustekinumab was installed at a later phase of the disease, when brain white matter lesions had already been formed, the efficacy was much lower, although the activity of the lesions (inflammation and enlargement) was completely suppressed (‘T Hart et al., 2005). We therefore hypothesized that during the course of the disease, a second pathogenic mechanism might be activated. A possible trigger may be the release of self antigens from lesions inflicted by the concerted action of Th1 cells and autoantibodies that we discussed above (‘T Hart et al., 2009). The existence of a second EAE perpetuation mechanism could indeed be confirmed (Kap et al., 2008).

### The EAE progression pathway

In monkeys with a relatively rapid EAE progression T cell reactivity against peptide MOG_34–56_ was detected (Kap et al., 2008). By serial magnetic resonance imaging (MRI) it was observed that the appearance of this reactivity occurs side-by side with a typical late-stage event in the rhMOG/CFA model, namely the induction of demyelination in the cortical gray matter (‘T Hart et al., 2004). We identified the phenotype, fine-specificity and MHC restriction of the MOG_34–56_ responsive T cell fraction via carboxyfluorescein succinimidyl ester (CFSE) vital dye dilution. The main proportion of the anti-peptide reactivity was found in a subpopulation of CD3+CD4+CD8α+CD56+CD28−CD27+CD16−CCR7− T cells (Kap et al., 2008; Jagessar et al., 2012b). The main functional characteristics of the cells were high production of IL-17A and specific cytotoxicity toward EBV-transformed B cells presenting peptides derived from MOG_34–56_. Based on the similarity of this phenotype and function with a subset of NK cell related cytotoxic T cells in the human immune system (Mazzarino et al., 2005) we proposed to name this subset natural-killer-cytotoxic T lymphocytes (NK-CTL). The specificity of these core pathogenic T cells was defined at MOG_40–48_ and the presenting MHC molecule as Caja-E (Jagessar et al., 2012b). In the IPD-MHC database (http://www.ebi.ac.uk/ipd/mhc/nhp/index.html) only two Caja-E alleles are published, indicating that, just like the human ortholog HLA-E, Caja-E is oligomorphic. The only difference between the two alleles is a single amino acid at position 123 (V or L) located outside the peptide-binding cleft. The expression of Caja-E within the marmoset brain was confirmed (Jagessar et al., 2012b).

We observed that the NK-CTL is directly activated in monkeys immunized with peptide MOG_34–56_ in incomplete Freund’s adjuvant (MOG_34–56_/IFA) (Jagessar et al., 2010). This exciting new model shows remarkable pathological similarity with progressive MS, in particular the presence of widespread demyelination in the cortical gray matter. Intriguingly, IgM and IgG antibodies binding ELISA plate-bound rhMOG, which is a requirement for their capacity to mediate demyelination (Menge et al., 2007), are not detectable in this model. Neither were such antibodies found in the circulation, nor was deposition of antibody and complement within the CNS detectable. For this reason we held the autoreactive cytotoxic T cells responsible for the cortical demyelination (Jagessar et al., 2012b).

Are these findings relevant for MS? It has been documented that the immune repertoire of MS patients is significantly enriched with high avidity CD4+ T cells against MOG_34–56_ (Bielekova et al., 2004). Others showed that the MS repertoire contains HLA-E restricted cytotoxic T cells that are capable to kill oligodendrocytes. These cytotoxic T cells display remarkable similarities with the Caja-E restricted cytotoxic T cells that drive MOG_34–56_/IFA induced EAE in marmosets (Broux et al., 2012; Zaguia et al., 2013). The specific epitopes of the Th1 cells that drive the initiation pathway (MOG_34–36_) and of the CTL that drive the progression pathway (MOG_40–48_) are juxta-positioned in an evolutionary highly conserved area (20–50) of the MOG extracellular domain. The only difference between evolutionary distinct species is at position 42, which is occupied by serine (S42 in MOG from mouse, rat, marmoset) or proline (P42 in MOG from man, rhesus, or cynomolgus macaque) (Table 2).

**Table 2:** **Alignment of amino acids sequences of the myelin oligodendrocyte glycoprotein extracellular domain (MOG1–125)**.

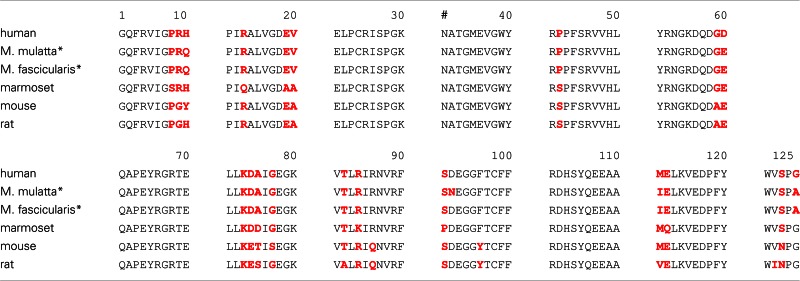

*MOG sequences were down-loaded from http://www.uniprot.org/uniprot/?query=myelin+oligodendrocyte+glycoprotein&offset=25&sort=score. All positions where there is variation between the species are marked bold and in red. The two identified dominant T cell epitopes identified in marmosets (for the CD4 Th1: MOG24–36 and for the CD4+CD8+CD56+ NK-CTL: MOG40–48) are juxtaposed in the highly conserved domain MOG21–50. The only N-glycosylation site in MOG is also located in this domain, namely Asn31 (#)*.

In summary, the rhMOG/CFA marmoset EAE model illustrates the difficulties encountered in the translation of therapeutic principles from the EAE model to the MS patient. The EAE initiation pathway in marmosets recapitulates classical mouse EAE models. Despite the highly relevant pathogenic role of the IL-12/IL-23 axis in mouse EAE models (Gran et al., 2004), which was reproduced by the promising efficacy of the anti-IL-12p40 mAb ustekinumab in the marmoset EAE model, the ustekinumab mAb lacked efficacy when tested in RRMS (‘T Hart et al., 2005; Segal et al., 2008). A possible explanation for this discrepancy may be that the mouse EAE-like initiation pathway has long passed when the diagnosis MS is first made. The question is therefore warranted whether the MOG34–56/IFA induced marmoset EAE model more closely resembles established MS.

## A Crucial Role of CD20+ B Cells in the Marmoset EAE Model

**Figure 1:**
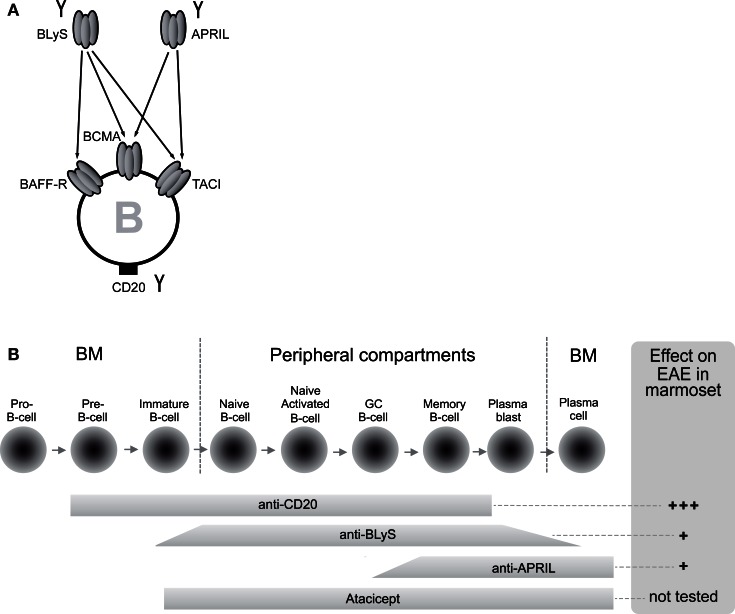
**Differential mechanisms and distinct EAE outcome of B-cell directed therapies. (A)** B-cell targeting therapies tested in the marmoset EAE model are neutralizing monoclonal antibodies (mAbs) against BLyS and APRIL and a depleting mAb against CD20 (indicated with the symbol Y). BLyS and APRIL are related cytokines expressed by myeloid and lymphoid cells with a specific action on B cells. B cells express three types of receptors; BAFF-R is specific for BLyS, whereas BCMA and TACI bind both factors. **(B)** Since the target of anti-CD20 mAbs is expressed throughout B-cell development, with the exception of pro-B-cells and plasma cells at the extreme ends, anti-CD20 mAb depletes a wide range of B cells. The narrower activity window of neutralizing antibodies against BLyS or APRIL or a soluble form of TACI (atacicept) shows partial overlap with the expression of CD20.

Several B-cell targeting therapies are now under development for therapeutic application in human autoimmune diseases (review: Davidson, 2010; Barun and Bar-Or, 2012). The specificities include CD20, a broadly expressed surface marker in the B-cell lineage, and the cytokines BlyS and APRIL, which exert overlapping activities on the survival and development of B cells (Figure 1).

Clinical trials with a chimeric anti-CD20 mAb (rituximab) show remarkable beneficial effects on clinical and MRI parameters in RRMS, subgroups of primary progressive MS, and in opticospinal disease (Barun and Bar-Or, 2012). Administration of anti-CD20 mAb induced rapid, profound, and long-lasting (months) depletion of B cells from blood and, albeit less profound, from the cerebrospinal fluid (Cross et al., 2006). However, the mechanisms underlying the remarkable and unexpected beneficial effects of B-cell depletion in MS are incompletely understood.

Studies in mouse EAE models show that B cells have a variety of effects, which depend on the stage of the disease. The production of anti-myelin autoantibodies that mediate demyelination via the activation of ADCC and CDC has already been mentioned. A most compelling set of data was reported by Matsushita et al. (2008) who observed in B6 mice that early depletion of B cells inhibited EAE due to abrogation of autoantibodies, whereas late B-cell depletion can augment EAE severity due to the depletion of IL-10+ regulatory B cells. Another study showed that the effect of B-cell depletion varies between EAE models (Weber et al., 2010). In B6 mice immunized with recombinant mouse MOG protein in CFA, B-cell depletion suppressed EAE, by inhibiting the induction of cellular (Th1, Th17) and humoral (autoantibody) autoimmune reactions. In this model, B cells are activated and they process and present MOG to the pro-inflammatory T cells. By contrast, in B6 mice immunized with MOG35–55/CFA depletion of B cells aggravated EAE, possibly because regulatory T cell activity was impaired.

For the preclinical efficacy studies in the marmoset EAE model we used HuMab7D8, a clonal variant of the fully human anti-CD20 IgG1κ mAb ofatumumab (Bleeker et al., 2008). Ofatumumab is effective in MS (Barun and Bar-Or, 2012) and HuMab7D8 cross-reacts well with marmoset B cells (Kap et al., 2010). The mAb was successfully tested in the rhMOG/CFA model, where anti-MOG autoantibodies have a pathogenic contribution, and in the MOG34–56/IFA model, where anti-rhMOG autoantibodies are not found (see below). All studies were designed to avoid interference with the immunization. Hence, the mAb treatment was started well after the EAE induction, i.e., at post-sensitization (psd) day 21. At this stage the autoimmune process is already ongoing, as illustrated by the presence of anti-rhMOG IgG in the serum (Kap et al., 2010).

### HuMab7D8 in the rhMOG/CFA model

The anti-CD20 mAb induced profound depletion of CD20+ B cells from peripheral blood, lymphoid organs, and prevented B-cell entry into the brain (Kap et al., 2010, 2011). The usually observed progressive increase of serum anti-rhMOG IgG levels measured in the control group was almost immediately stopped in treated monkeys illustrating the brisk systemic effect of the mAb. The clinical effect was remarkable as none of the mAb-treated monkeys developed evident clinical EAE; the formation of demyelinated lesions in the gray and white matter of brain and spinal cord was also dramatically reduced. Intriguingly, also the production of T cell and macrophage cytokines that have a key function in the EAE pathogenesis, such as IFNγ, IL-17A, TNF-α, and IL-7, was impaired.

### HuMab7D8 in the MOG_34–56_/IFA model

As discussed above, the pathogen-educated immune system of marmosets contains effector memory T cells, which express CD4, CD8α, CD27, and CD56, but are negative for CD28 and CCR7 (Jagessar et al., 2012a). These T cells are the central drivers of inflammation and demyelination in the EAE model induced with MOG_34–56_/IFA (Jagessar et al., 2010). The model recapitulates clinical and pathological features of the more complex rhMOG/CFA EAE model, although it was induced without ligands for innate antigen receptors in the immunizing inoculum and anti-rhMOG autoantibodies could not be detected in the sera from immunized monkeys (Jagessar et al., 2010). Although the exact pathogenic mechanisms via which the autoreactive T cells elicit EAE pathology and symptoms in this model have only partially been resolved, a pathogenic role of the high production of IL-17A, and the Caja-E restricted cytotoxicity toward EBV-transformed B cells presenting the MOG_40–48_ core epitope is likely (Jagessar et al., 2012b). Strikingly, weekly administration of HuMab7D8 from psd 21 onward completely abrogated EAE development in this model. The activity of the antibody was reflected by a variable effect on the T cell compartment, measured at necropsy (Jagessar et al., 2012a). The *ex vivo* proliferation of blood mononuclear cells (PBMC) induced with MOG_34–56_ was not significantly different between the control and anti-CD20 treated monkeys. Whereas mRNA transcript levels of perforin and granzyme B, both markers of the NK-CTL, were reduced in axillary lymph nodes (ALN), we observed no changes in spleen. By contrast, mRNA levels of IFNγ and IL-17A were reduced in spleen but increased in the ALN. These data suggest that the effect of B-cell depletion may not be prevention of T cell activation. One possible explanation may be that the distribution of the pathogenic T cells over the secondary lymphoid organs and the target organ may be altered by the depletion of B cells. We are therefore performing a systemic analysis of T cell activity profiles in the whole lymphoid compartment. However, these data are not yet available.

The results of both studies warrant the conclusion that B cells have a key pathogenic role in the marmoset EAE model. The pathogenic function of the B cells includes, but is likely not confined to, the activation of Caja-E restricted CD3+CD4+CD8α+CD56+ effector memory cytotoxic T cells that induce MS-like pathology in cortical gray matter.

## Moderate Efficacy of Anti-BLyS and Anti-APRIL mAbs in rhMOG/CFA EAE

The obvious key question in this context is whether all CD20+ B cells are capable to activate the core pathogenic subset of cytotoxic T cells or whether this capacity is confined to a certain B cell subpopulation. To address this issue, we tested the efficacy of two neutralizing mAbs against the growth and survival factors BLyS and APRIL, which B cells need for their survival and development (see Figure 1).

The B-cell cytokines BLyS and APRIL are members of the TNF superfamily, expressed by a wide range of myeloid and lymphoid cells, B cells included (Rickert et al., 2011). The two cytokines relay their stimulatory signals to B cells via three different receptors: TACI and BCMA bind BlyS and APRIL, while BR3 binds only BLyS (Figure 1A). The co-stimulatory signals relayed to B-cell subsets enhance their survival and development. Deregulation of BLyS has been associated with autoimmune disease in experimental models and human patients, such as with SLE, RA, or Sjögren syndrome (Rickert et al., 2011). The BLyS inhibiting mAb belimumab has recently received FDA approval for treatment of SLE (Liu and Davidson, 2011). Atacicept, a recombinant fusion protein composed of human Ig-Fc fragment with the ligand binding unit of TACI, the shared joined receptor of BLyS and APRIL, has been evaluated in MS. However, two trials with atacicept had to be stopped due to an unexpected increase of inflammatory activity on brain MRI scans in one of the trials (Hartung and Kieseier, 2010).

The efficacy testing of anti-BLyS and anti-APRIL mAbs in the rhMOG/CFA EAE model showed profound depletion of peripheral B cells, with the exception of a subset of CD40^*high*^ B cells. Intriguingly, this subpopulation was depleted in the monkeys treated with anti-CD20 mAb (Jagessar et al., 2012a). However, while the anti-CD20 mAb treatment completely prevented EAE development, all monkeys treated with anti-BLyS and anti-APRIL mAb developed clinically evident EAE albeit with a delayed onset of about 2 weeks (Jagessar et al., 2012b).

How can the discrepant effects of anti-CD20 and anti-BlyS or APRIL mAb be explained? The shortage of cross-reactive antibodies with which B-cell subsets can be phenotyped has thus far prohibited an in depth analysis of changes in the B-cell compartment induced by the different treatments. In the context of the above-mentioned systems analysis we are developing such markers.

One of the major differences between conventionally housed NHP and SPF rodents is that NHP are infected with similar herpes viruses as those infecting humans. The marmoset equivalent of human EBV, CalHV3, is a B-cell transforming lymphocryptovirus (Cho et al., 2001). It is of note that EBV-infected B lymphoblastoid cell lines of marmosets are CD20+, express high CD40 and produce BLyS, but are insensitive to BLyS inhibition by the anti-BLyS mAb (Jagessar et al., 2013). This observation raised the question whether the distinct effects of anti-CD20 and anti-BLyS mAb could be explained by differential depletion of CalHV3-infected B cells. We used a qPCR developed in-house for quantification of CalHV3 DNA copy numbers developed in lymphoid organs. Spleen and lymph nodes from non-treated EAE marmosets and anti-BLyS treated EAE marmosets contained high CalHV3 titers, whereas these were reduced>90% in EAE marmosets treated with anti-CD20 mAb (Jagessar et al., 2013). This observation indeed suggests that CalHV3 containing B cells are depleted in marmosets treated with anti-CD20 mAb, but not or to a lesser extent in marmosets treated with anti-BLyS mAb. It is tempting to speculate that the discrepant efficacy of anti-CD20 mAbs and atacicept in MS may be based on a similar mechanism.

## Translation to MS

We postulate that the efficacy of treatment with B-cell targeting mAbs in the marmoset EAE model is related to the depletion of CalHV3-infected B cells. The direct implication of this postulate is that B cells infected with the virus have a crucial pathogenic role in the EAE model.

An important question is the relevance for the treatment of MS. There is a vast body of literature data supporting the association of EBV infection with MS susceptibility (Christensen, 2007; Lunemann and Munz, 2009; Pender, 2011), but the underlying mechanism(s) remain poorly understood. Intriguingly, MS-like diseases associated with γ-herpesvirus infection were recently reported in mice (Casiraghi et al., 2012) and in macaques (Axthelm et al., 2011). The marmoset EAE model suggests that infection with the EBV-counterpart CalHV3 renders B cells capable of recruiting autoreactive cytotoxic T memory cells from the normal repertoire. Data obtained both in rhesus monkey and marmoset EAE models indicate that the original trigger of these memory cells may be another herpes virus, cytomegalovirus (CMV). Cytotoxic T cells isolated from rhesus monkeys sensitized against MOG_34–56_ were found to proliferate *ex vivo* against peptide 981–1003 from the UL86 ORF-encoded major capsid protein, an immunodominant antigen of CMV (Sylwester et al., 2005). We observed that the CMV peptide and MOG_34–56_ share a cross-reactive mimicry epitope (MOG_39–48_) (Brok et al., 2007). The cytotoxic T cells that mediate MS-like pathology and disease in the MOG_34–56_/IFA induced marmoset EAE model recognize the MOG_40–48_ epitope (Jagessar et al., 2012b). It is therefore tempting to speculate that the autoreactive CTL that mediate EAE in NHP may be recruited from the sizeable natural repertoire of effector memory T cells directed against CMV. Indeed, recent work from Broux and Hellings shows an important pathogenic role of MOG-specific CD4+CD28−CX3CR1+ T cells in MS (Broux et al., 2012). These T cells are thought to sustain chronic inflammatory pathology. As reviewed elsewhere, CMV may be a main driver of the progressive expansion of the CD28^*null*^ subset in the aging immune system (‘T Hart et al., 2013). Interestingly, in an old study the presence of CMV-reactive T cells within MS brain lesions was already reported (Scotet et al., 1999). The marmoset model thus supports the possibility that MS susceptibility may be associated with two herpes viruses, namely EBV and CMV.

What could be the underlying mechanism? During the co-evolution with their natural host, CMV and EBV have developed an elaborate repertoire of strategies to avoid detection by the immune system. One of the escape strategies employed by CMV is minimizing exposure of viral antigens via impairment of the cell surface expression of peptide-loaded classical MHC class Ia molecules (HLA-A, -B, -C) with collateral upregulation of peptide-occupied MHC class Ib (HLA-E/gpUL40) to resist attack by natural killer cells (Braud et al., 2002). A similar evasion mechanism is employed to avoid immunity at the feto-maternal interface (Hunt, 2006), toward cancers (Wischhusen et al., 2007) and possibly also within immuno-privileged organs such as the brain (Jiang and Chess, 2000). It is thus tempting to speculate that mechanisms developed by the immune system to combat immune escape of CMV may be involved in the induction and/or progression of autoimmune disease. Is the risk to develop autoimmunity the price we have to pay for effective protection against the exacerbation of latent herpesvirus infection?

A T cell subset in the human immune system with a presumed role in the control of CMV is NK-CTL (Moretta et al., 2003; Mazzarino et al., 2005). NK-CTL are CD8β+ CTLs that recognize viral peptides presented by the non-classical MHC-1b molecule HLA-E. The human immune repertoire contains a sizeable number of such HLA-E restricted NK-CTL, which seems to increase with aging (Mazzarino et al., 2005). It is intriguing that these HLA-E restricted NK-CTL (CD8β+CD45RA+CD27−CD28−CD56+CCR7−) (Mazzarino et al., 2005) resemble phenotypically the Caja-E-restricted CTL (CD4+CD8α+CD45RO−CD27+CD28−CD56+CCR7−) that mediate EAE in the MOG_34–56_/IFA induced marmoset EAE model (Jagessar et al., 2012b). The marmoset cells may also be equivalent to the already mentioned CD4+CD56+ cytotoxic T cells found in the MS repertoire, which kill human oligodendrocytes via an HLA-E restricted mechanism (Zaguia et al., 2013). At this moment we cannot distinguish between these two options as we could only use an anti-CD8α mAb for phenotyping of the marmoset cells. The mAb does not discriminate between CD8αβ heterodimer and CD8αα homodimer molecules. The latter is transiently expressed on αβTCR+ T memory effector cells (Madakamutil et al., 2004) as a ligand of the thymic leukemia antigen TL, a non-classical MHC molecule that does not bind CD8αβ (Leishman et al., 2001).

Although various APC types are capable of presenting soluble antigens via MHC-I to CD8+ T cells, DC seem to be the main APC equipped for this task, at least in mice. Also B cells can cross-present soluble antigen via MHC-I *in vitro*, albeit only under particular conditions, including the presence of danger signals relayed via TLR9 (Jiang et al., 2011), or after infection with EBV (Jagessar et al., 2013). Important in this context is that latently EBV-infected B cells escape immunity by down-regulating classical MHC class Ia (HLA-A, -B, -C) and upregulating MHC class Ib (HLA-E, -G) molecules (Dutta et al., 2006). It may thus be a reasonable assumption that latently EBV-infected B cells have the capacity to present *in vivo* soluble autoantigen via HLA-E to NK-CTL. This has actually been shown in marmosets (Jagessar et al., 2013).

Considering the elusive association of EBV with MS the question has been raised how the discrepancy between the high prevalence of EBV infection (>99%) and the low prevalence of MS (<1/1000 young adults) in the human population can be explained (Christensen, 2007). It has been well established that B cells are particularly effective APC when they can take up soluble antigen via their specific antigen receptor and present it to T cells of the same specificity (Lanzavecchia and Bove, 1985). As reasoned above, for acquiring the capacity to present soluble antigen via MHC class I to CD8+ T cells, the antigen-specific B cell needs to be infected with EBV. An important variable is thus the chance that an antigen-specific B cell is infected with EBV. In healthy individuals the frequency of EBV-infected B cells has been determined at 1–50 per 10^6^ (Khan et al., 1996). Although the number of B-cell clones differing in antigen-specificity has been estimated at around 10^9^, the size of individual clones varies enormously, ranging between 100 cells per clone against new antigens (Quintans and Lefkovits, 1974) to several thousands for autoantigen-specific B-cell clones in an autoimmune disease patient (Logtenberg et al., 1986). Taken together, these findings suggest that the chance that an autoreactive B-cell clone gets infected with EBV, thereby acquiring the capacity to cross-present soluble antigen to CD8+ T cells, may be rather low.

In conclusion, we propose that two herpes viruses may be implicated in the pathogenesis of MS.

### Cytomegalovirus

HLA-E-restricted NK-CTL play an important role in the lifelong combat with latent CMV infection. These cells form a broad reservoir of highly reactive T cell specificities that progressively expands with aging (Sylwester et al., 2005). At least one of the specificities can be stimulated by a cross-reactive peptide (MOG_40–48_) shared between a viral antigen (CMV UL86) and an autoantigen unique to the CNS (MOG).

### Epstein Barr virus

We also postulate that this mimicry epitope (MOG_40–48_) can be cross-presented by EBV-infected B cells via HLA-E to the HLA-E restricted NK-CTL. When NK-CTL are activated via this route, they can find their way into the CNS, where they either directly or indirectly elicit inflammation and demyelination in white and gray matter of brain and spinal cord. However, it is unknown at this stage whether the NK-CTL express the fractalkine receptor CX3CR1, which is prominently expressed on CD4+CD28− cytotoxic T cells in MS (Broux et al., 2012).

The therapeutic perspective and testable hypothesis following from this postulate is that therapeutics specifically targeting EBV-transformed B cells are a safe and effective treatment for MS.

## Conflict of Interest Statement

The authors declare that the research was conducted in the absence of any commercial or financial relationships that could be construed as a potential conflict of interest.
